# Helical TomoTherapy for locally advanced or recurrent breast cancer

**DOI:** 10.1186/s13014-016-0736-1

**Published:** 2017-01-28

**Authors:** M. N. Duma, C. Heinrich, C. Schönknecht, B. Chizzali, M. Mayinger, M. Devecka, S. Kampfer, S. E. Combs

**Affiliations:** 1 Department of Radiation Oncology, Klinikum rechts der Isar der Technischen Universität München, Ismaninger Str. 22, 81675 München, Germany; 2 Praxis für Strahlentherapie, Hausham, Germany; 3 Institute of Innovative Radiotherapy (iRT), Helmholtz Zentrum München, München, Germany

**Keywords:** Helical tomo therapy, Breast cancer, Reirradiation, Chemoradiation, Acute toxicity

## Abstract

**Purpose:**

We report our experience of using helical tomotherapy (HT) to treat large and irregular shaped loco-regional advanced breast cancer target volumes embracing various organs at risk.

**Patients and methods:**

We retrospectively analyzed 26 patients treated for very large, irregular shaped breast cancers. Patients were treated either with the intent to achieve local control in a primary setting (*n* = 14) or in a reirradiation setting (*n* = 12). The recurrence group was heavily pretreated with systemic therapy. Tumors were characterized by wide infiltration of the skin, encompassing mostly a complete hemithorax. The primary group underwent irradiation of supraclavicular, infraclavicular, axillary and parasternal lymphonodal region. Radiotherapy was combined with chemotherapy (*n* = 11). We assessed the PTV volume and its craniocaudal extension, the dose to the organs at risk, acute toxicity and survival.

**Results:**

Median PTV was 2276 cm^3^ (1476–6837 cm^3^) with a median cranio-caudal extension of 28 cm (15–52 cm). The median dose to PTV was 40 Gy (32–60Gy). HT could be carried out in all patients without interruption. The acute toxicities were mild to moderate. The median LRFS and OS after radiotherapy was 21 and 57 months for the primary group versus 10 and 11 months for the recurrence group. Median PFS was 18 months (primary group) and 7 months (recurrence group).

**Conclusions:**

HT is feasible for advanced thorax embracing target volumes with acceptable acute toxicity. Both curative and palliative indications can be considered good indications based on treatment volume and anatomical constellation.

## Introduction

The standard of care for the treatment of local breast cancer recurrence is surgery. High local control rates can be achieved with mastectomy after primary breast conserving therapy [1].

In case of inoperable locally advanced chest wall recurrences the standard treatment is radiotherapy [2]. However in most cases whole breast irradiation after breast conserving surgery was performed beforehand. Thus, exposure of preirradiated normal tissue (skin, soft tissues, bones, lungs, heart) limits the reirradiation options.

Radiotherapy for large and complexly shaped tumor volumes in case of local recurrence or primary locally advanced tumor is often not reasonably achievable with conventional 3D conformal radiotherapy (3D-CRT). This is mostly due to the fact, that the tumor partly embraces organs at risk (OAR) such as the lungs or heart [3]. Planning studies for the use of helical tomotherapy (HT) for breast cancer treatment have been shown to improve conformality to the tumor bed, while sparing organs at risk (OARs). While HT has been studied after breast conserving surgery in the context of partial breast radiation [4, 5], whole breast radiation [6] and loco regional nodal radiation [3] only few data are available on the feasibility, acute side effects and outcome of helical tomotherapy in the context of large chest wall recurrences and reirradiation and primary locally advanced tumor volumes [7, 8].

The following retrospective analysis presents a single university center experience with treatment of advanced breast cancer with/without chemotherapy using HT.

## Material and methods

### Patient characteristics

From April 2007 to August 2011, 26 patients (age range: 34–79 years) were treated for very large, irregular shaped local recurrences (*n* = 12) or for locally advanced breast cancer in a primary setting (*n* = 14) with HT. Table 1 depicts the patients' characteristics. HT was at the given time the only machine available in our department that could perform IMRT and (daily) CT based IGRT and treat extensive volumes.

#### Reccurence group

**Fig. 1:**
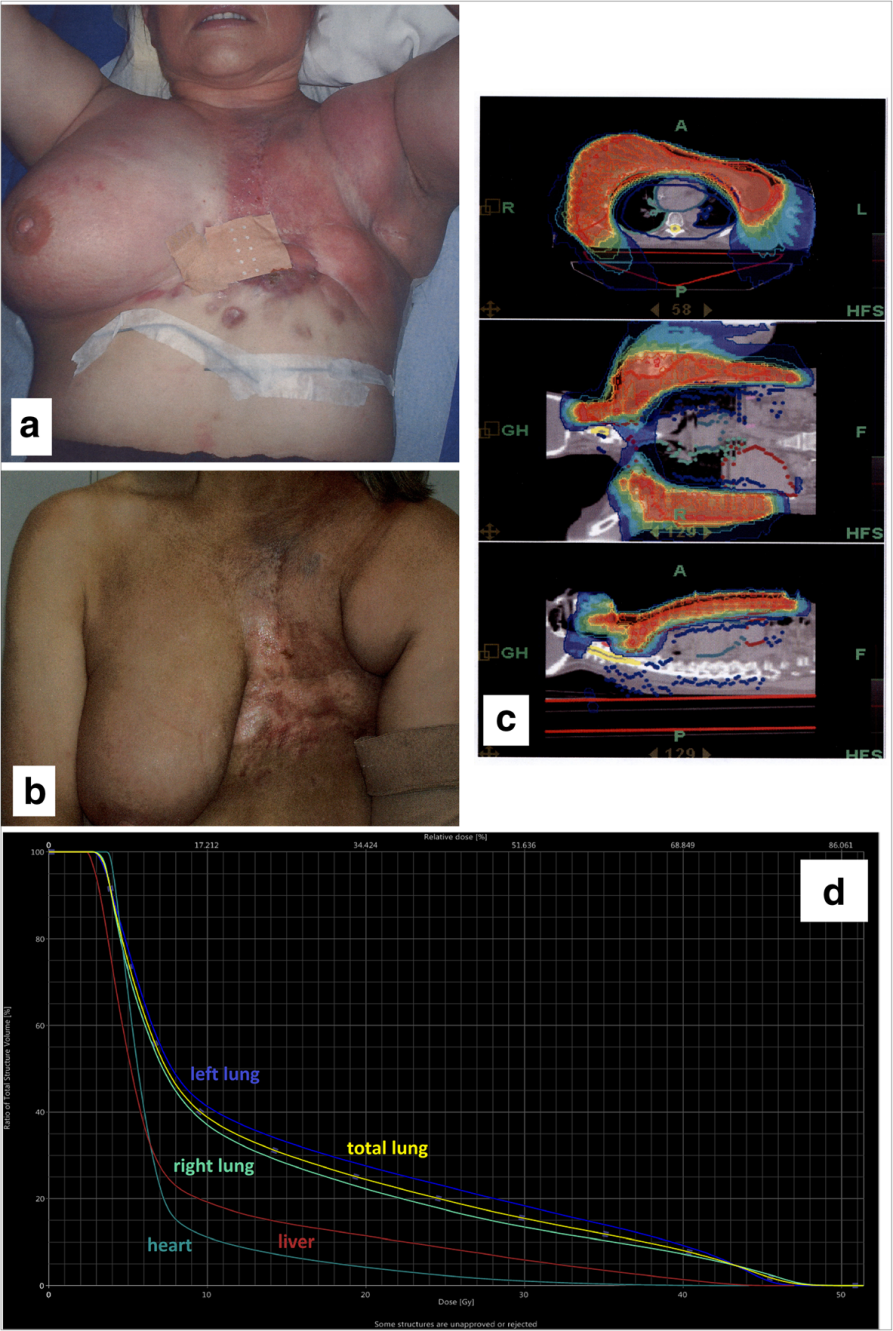
Patient from the recurrence treatment group **a**: clinical appearence at the start of HT; **b**: clinical appearence 6 weeks after HT; **c**: HT treatment plan; **d** DVH

All patients in the recurrence group were treated with a palliative intent. Tumors in this group were characterized by wide infiltration of the skin and subcutaneous tissue as well as extensive lymph node metastases. Palliative treatment was offered for ulcerating tumors, pain and/or neurological symptoms in patients with extensive bulky disease. Six patients received an additional individualized boost treatment with neutrons. Neutrons are used on a routine basis in our department in a third or higher reirradiation setting of extended cutaneous/subcutaneous metastasis. Ten patients had distant metastases and ongoing systemic therapies. Most patients were heavily pretreated and systemic therapies varied depending on the pretreatment. For nine patients it was necessary to continue dose-reduced chemotherapy during reiradiation because of systemic progression. Nine of the 12 patients (75%) in the recurrence group had initially received breast conserving surgery followed by whole breast radiotherapy (WBRT). Three patients had radical mastectomy. Figure 1 depicts a typical patient from the recurrence group.

**Fig. 2:**
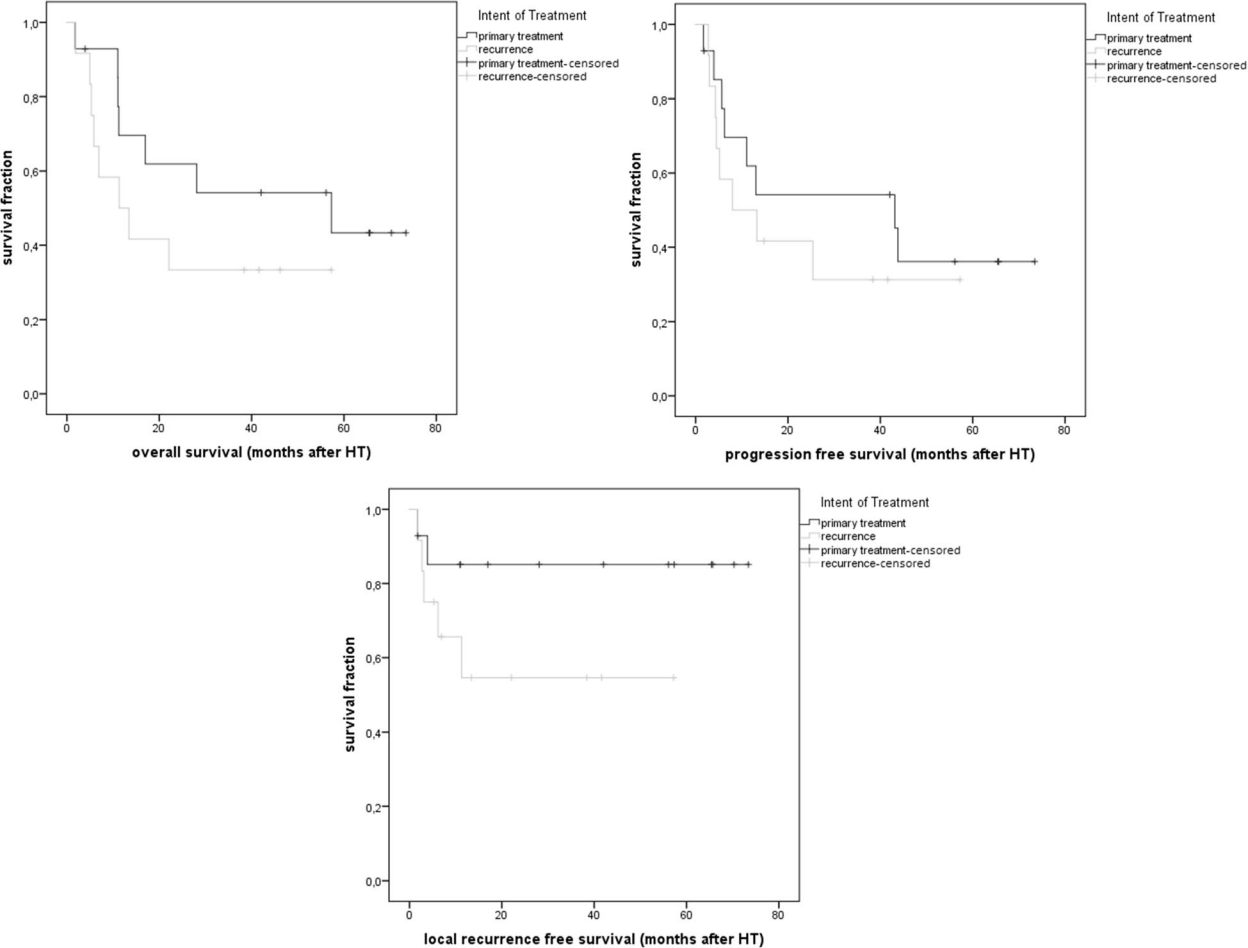
Overall survival (OS), progression free survival (PFS) and local recurrence free survival (LRFS) after HT for each group

#### Primary setting group

Patients in primary setting had locally advanced tumors with extensive involvement of axillary lymph nodes (bulky disease) and macroscopic parasternal lymph nodes or were at high risk of parasternal lymph nodes metastases. One patient underwent polychemotherapy without surgery. 8 of 14 (57%) patients underwent radical mastectomy and the remaining 6 (43%) patients received breast conserving surgery. Nonetheless in 3 of these 6 patients radical mastectomy was necessary after a prior breast conserving attempt. Seven of the 14 (50%) patients had positive resection margins (5 pts.- R1 resection, 2 pts.- R2 resection) after radical mastectomy. Five of the 11 (45%) patients after radical mastectomy had extensive lymphangiosis carcinomatosa.

### Treatment planning and delivery

Before treatment, each patient underwent a planning computer tomography (CT) scan (Siemens Somatom; Siemens Inc., Erlangen, Germany) with an axial slice thickness of 3 mm. A vaccum bag (Bodyfix, Medical Intelligence, Schwabmuenchen, Germany) was used for immobilization. The contouring of the PTV and the OARs was performed on the Oncentra Master-Plan system (Nucletron B.V., Veenendaal, The Netherlands) and iPlan (Brainlab, Feldkirchen, Germany). Treatment planning was done using Tomotherapy HI-Art System® (Accuray Inc., Sunnyvale CA, USA). The prescribed dose to the PTV was dependent on previous irradiation regimes and ranged from 32 to 60 Gy with a median PTV dose of 40 Gy. Organs at risk (OARs) were contoured according to internal guidelines and constraints were set according to Emami et al. [9]. In case of reirradiation, the spinal cord biological equivalent dose was calculated according to Nieder et al. [10]. All patients underwent daily image guided radiotherapy (IGRT). After acquisition and reconstruction, the daily megavoltage CT scans (MVCT) were automatically registered, by choosing the bone and tissue algorithm provided by TomoTherapy, to the planning CT. To account for the best positioning of the patient, every automatic registration was controlled and corrected by experienced staff members before treatment.

### Outcome

Acute toxicity was assessed using the Radiation Therapy Oncology Group/National Cancer Institute Common Toxicity Criteria (RTOG/NCI CTC), version 3.0, morbidity scales. Acute toxicity was assessed every week during HT and 6–8 weeks after treatment. The planned follow up visits were: 6–8 weeks after HT, 3, 6 and 12 months after HT and thereafter once a year. Most clinical follow up data presented herein on local control was collected from the first visit 6–8 weeks after treatment. Many patients were then lost to follow up and there is not enough data available to reliably assess the late side effects. Treatment response was measured by clinical examination and radiological findings. Complete response was defined as the clinical disappearance of all irradiated target lesions. A partial response was defined as a decrease of more than 50% of the target lesion. Stable disease was defined as a decrease less than 50% or no change. Progressive disease was defined as an increase of target lesions or appearance of new lesions (“in field” or throughout the body). Data for PFS, LRFS and OS was collected from different sources (patient records, data from primary oncologists, the Munich Cancer Registry). Data are reported by means of descriptive statistics and Kaplan Meier survival function.

## Results

### Treatment parameters

HT could be carried out to the planned cumulative dose in all patients without interruption.

Table 2 depicts the treatment parameters for both treatment groups.

**Table 1:** Patients’ characteristics

	All patients	Recurrence Treatment Group	Primary Treatment Group
Descriptive characteristics			
n	26 (100%)	12 (100%)	14 (100%)
Median age (range)	63 (31–75)	52 (31–75)	69 (62–74)
Median Karnofsky Performance Score (range)	90% (70–90%)	90% (70-90%)	90% (80–90%)
Distant metastases	13 (50%)	6 (50%)	7 (50%)
Histology			
Invasiv ductal	21 (81%)	9 (75%)	12 (86%)
Invasiv lobular	4 (15%)	2 (17%)	2 (14%)
Angiosarcoma	1 (4%)	1 (8%)	−
Side			
Left side	12 (46%)	6 (50%)	6 (43%)
Right side	10 (38%)	4 (33%)	6 (43%)
Bilateral	4 (15%)	2 (17%)	2 (14%)
Symptoms at radiotherapy start			
Exulcerating/painful tumour	8 (31%)	6 (50%)	2 (14%)
Extensive lymph node metastases with lymphedema	9 (35%)	5 (42%)	4 (29%)
Previous treatments			
Breast conserving surgery	15 (58%)	9 (75%)	6 (43%)
Median Dose WBRT	−	60 Gy (50.4–66 Gy)	−
Prior radical mastectomy	14 (54%)	3 (25%)	11 (79%)
Prior polychemotherapy	10 (38%)	6 (50%)	4 (29%)
Prior adjuvant endocrine treatment	15 (57%)	4 (33%)	11 (79%)

Depicted are absolute values or median values with range

**Table 2:** Treatment Parameters

	Recurrence Treatment Group (*n* = 12)	Primary Treatment Group (*n* = 14)
Concurrent chemotherapy	3 patients vinorelbin 4 patients capecitabin 1 patient sunitinib 1 patient doxorubicin	1 patient vinorelbin 1 patient capecitabin
PTV localization/extension^a^		
a) Ipsilateral chest wall	2 patients	2patients
b) More than 1/2 of the thoracic circumference plus loco-regional lymph nodes	4 patients	9 patients
c) More than 2/3 of the thoracic circumference plus loco-regional lymph nodes	6 patients	3 patients
d) Extranodal metastasis within the PTV	1 patient (pleura)	2 patients (bone lesions)
e) Extensive skin metastases	4 patients	
PTV volume (cm^3^)	2984 (1457–6837)	1330 (520–6623)
Cranio-caudal PTV extension (cm)	30 (19–52)	28 (18,2–43,5)
Median total dose	40 Gy (32–60 Gy)	50 Gy (40–60 Gy)
Median dose per fraction	2 Gy (1,8–3,0 Gy)	2,0 Gy (1,5–2,24 Gy)
Simultaneous integrated boost	2 patients (50.4/56 Gy with single doses of 1.8/2.0Gy; 55,8/60,1 Gy with single doses of 1.8/1.94 Gy)	3 patients (45/50 Gy with single doses of 1.8/2.0 Gy; 45/50/56 Gy with single doses of 1.8/2.0/2.24 Gy; 46Gy/50 with single doses of 2.0/2.17 Gy)
Neutron boost	6 patients (4.6–12Gy)	−

^**a**^The PTV comprised more than a hemi-thorax in 13 and more than two thirds of the thoracic circumference in 9 patients

12 patients with extensive local recurrences received a total reirradiation dose which ranged from 32 to 60 Gy total dose (1.8–3 Gy daily) depending on palliative need, time between initial therapy and retreatment and initial irradiation dose. Two patients were treated with a simultaneous integrated boost, one patient with electron boost and six with neutron boost. The median interval between initial radiotherapy and reirradiation was 2.4 years (1–21 years). The median dose from the previous radiotherapy was 60 Gy (50.4–66 Gy). The 14 patients with primary locally advanced tumors received a total dose between 40 and 60 Gy. Three patients were treated with a simultaneous integrated boost.

The median mean dose (Dmean) to the heart in the primary and retreatment group was 13 Gy (1.5–34 Gy) and 12Gy (6–22 Gy) and the median Dmean to the lung was 21Gy (12–25.5 Gy) in the primary treatment group and 16Gy (5–20 Gy) in the retreatment group, respectively. Overall, the V5 was 95% (25–100%); the V20 18% (5–45%) and the V30 12%(1–27%). Overall 11 patients received a combined radiochemotherapy depending on pretreatment, systemic progression and kidney function (Table 2). Five patients received capecitabine (825 mg/m^2^ bid, including weekends), Four patients vinorelbine (25 mg/m^2^ 8q or 60 mg/m^2^14q), one patient doxorubicin (40 mg q28d) and one patient sunitinib (12,5 mg daily). Concurrent chemotherapy was well tolerated and could be carried out as planned during HT treatment without interruptions. Additionally, four patients continued endocrine treatment (two patients anastrozol, two patients letrozol).

### Acute side effects

The acute toxicity was mild to moderate measured weekly during therapy, 6–8 weeks and three to 4 months after treatment. Table 3 depicts the acute side effects.

**Table 3:** Acute side effects during treatment with HT and concurrent chemotherapy

Common Toxicity Criteria (CTC)	All patients	Recurrence Treatment Group	Primary Treatment Group
Radiotherapy	*n* = 26	*n* = 12	*n* = 14
Radiodermatitis °1	14 (53%)	6 (50%)	8 (57%)
Radiodermatitis °2	6 (23%)	4 (33%)	2 (14%)
Radiodermatitis °3	6 (23%)	2 (16.7%)	4 (28%)
Dysphagia °1	10 (%)	5 (42%)	5 (36%)
Lymphedema °3	3 (11%)	4 (33%)	1 (7%)
Fatigue	12 (46%)	5 (42%)	7 (50%)
Chemotherapy	*n* = 11	*n* = 9	*n* = 2
Leucopenia °1	6 (55%)	5 (55%)	1 (50%)
Leucopenia 2	5 (42%)	4 (44%)	1 (50%)
Anemia 1	6 (55%)	6 (67%)	−
Anemia 2	3 (27%)	2 (22%)	1 (50%)
Anemia 3	1 (9%)	−	1 (50%)
Hand-foot-syndrome	2 (18%)	2 (22%)	−

### Outcome

Overall, at the end of the treatment 23% (*n* = 6/26) patients had a local partial remission, 69% (*n* = 18/26) a stable disease, 7.7% (*n* = 2/26) a local progressive disease and 15% (*n* = 4/26) had already a systemic progression. Six to eight weeks after HT 50% (*n* = 6/12) of the retreatment group showed a local partial remission and 33% (4/12) showed a complete local remission “in field”. All four patients received a neutron boost.

Table 4 depicts the local outcome and the early side effects. Three to four months after HT 3 patients deceased and 6 patients were lost to follow up. Overall 18% (3/17) showed local progression and 29% (5/17) had systemic progression.

**Table 4:** Local outcome and palliative effects

	All patients	Recurrence Treatment Group	Primary Treatment Group
End of HT	*n* = 26	*n* = 12	*n* = 14
Local partial remission	6 (23%)	4 (33%)	2 (14%)
Local complete remission	−	−	−
Local stable disease	20 (76%)	6 (50%)	12 (85%)
Local progression	2 (8%)	2 (17%)	0
Systemic progression	4 (15%)	2 (17%)	2 (14%)
Acute symptom relief	7 (27%)	5 (42%)	2 (14%)
6–8 weeks after HT	*n* = 23	*n* = 12	*n* = 11
Local partial remission	8 (35%)	6 (50%)	2 (18%)
Local complete remission	4 (18%)	4 (33%)	−
Local stable disease	7 (30%)	−	7 (64%)
Local progression	1 (4%)	−	1 (9%)
Systemic progression	3 (13%	2 (17%)	1 (9%)
Lost to follow up	2	−	2
Deceased	1	−	1
3–4 months after HT	*n* = 17	*n* = 8	*n* = 9
Local partial remission	2 (11%)	−	2 (22%)
Local complete remission	−	−	−
Local stable disease	7 (42%)	3 (38%)	4 (44%)
Local progression	3 (18%)	1 (13%)	2 (22%)
Systemic progression	5 (29%)	4 (50%)	1 (11%)
Lost to follow up	6	2	4
Deceased	3	2	1

The median local recurrence free survival (LRFS) after radiotherapy was 21 months for the primary treatment group and 10 months for the recurrence group. Median overall survival (OS) after HT was 57 months (0–120 months) for the primary treatment group and 11 months (0.3–22 months) for the recurrence group. In the primary treatment group four patients died of systemic progress within the observing interval. In the recurrence group seven patients died of systemic progress within the observing interval. Median progression free survival (PFS) after HT was 43 months for the primary treatment group and 7 months for the recurrence group. Figure 2 depicts the overall survival (OS), progression free survival (PFS) and local recurrence free survival (LRFS) after HT for each group.

## Discussion

Patients with locally advanced breast cancer in the primary setting are managed in a multidisciplinary approach. Operable tumors may be managed with modified radical mastectomy followed by chemotherapy and locoregional radiotherapy. Inoperable tumors may be treated with neoadjuvant chemotherapy followed by operation and radiotherapy. Some departments perform combined treatment with hyperthermia. Postoperative radiotherapy (chest wall and regional lymphatics) decreases the risk of local recurrence after mastectomy and improves overall survival for patients with high risk of local recurrence [11–13]. Internal mammary and supraclavicular irradiation for breast cancer reduces breast-cancer mortality and improves disease-free survival and distant disease-free survival [14].

For patients whose tumors remain inoperable after neoadjuvant chemotherapy the management is less clear. Some studies have used radiochemotherapy or radiation alone performed with conventional radiation techniques as an attempt to downsize the tumor mass before surgery [15–17].

It is still unclear what is the best technique for treating extensive disease in breast cancer patients. Some plan comparison studies are available on this topic [3, 4, 18–21]. Zhang et al. evaluated the dosimetric benefit of volumetric modulated arc therapy (VMAT) on post-mastectomy left-sided breast cancer patients, with the involvement of internal mammary nodes [21]. VMAT achieves similar or superior target coverage and a better normal tissue sparing as compared to intensity-modulated radiotherapy (IMRT). Haciislamoglu et al. evaluated the dose distribution and homogeneity of four different types of IMRT (forward-planned IMRT, inverse-planned IMRT, HT and VMAT) in comparison with standard wedged tangential-beam 3D-CRT of the left breast in patients who had undergone lumpectomy [20]. All evaluated modalities provided adequate coverage of the whole breast. HT resulted in the lowest maximum doses delivered to the ipsilateral organs. Thus, if available, HT seems to be a good alternative. Chira et al. used neoadjuvant radiochemotherapy by helical tomotherapy for a group of 5 patients with locally advanced breast cancer [8]. All patients underwent mastectomy after radiotherapy. The preliminary results of Chira et al. showed that HT is feasible for neoadjuvant radiotherapy with acceptable toxicity profiles. Similarly, our patients treated in a primary setting with internal mammary and medial paraclavicular irradiation had low acute toxicities despite the very large treated volumes (maximum 6623 cm^3^). The median LRFS was 21 months and the median OS was 57 months.

Literature on reirradiation for locally advanced breast cancer recurrences with extensive skin infiltration and large bulky disease is sparse and not available on a systematic base concerning radiotherapy techniques or concurrent chemotherapy treatment. Some authors showed that reirradiation by 3D-CRT for patients with isolated local breast cancer recurrence should be considered as a salvage treatment with low to moderate toxicity and durable loco-regional control [7, 22–24]. Chatterjee et al. used HT for re-irradiation of three cases of supraclavicular disease from breast cancer. HT achieved a low dose to the brachial plexus without symptoms of brachial plexopathy and good local control of the supraclavicular disease [25]. Würschmidt et al. treated a heterogeneous group of 29 patients with reirradiation of locoregional recurrences of breast cancer [24]. They analyzed patients with operable (R1 or R0 resection) and non-operable recurrent breast tumors with and without distant metastases. However, most patients treated in this study had only a (extensive) thoracic wall recurrence, with just some patients with the axilla as simultaneous recurrence site. Thus the different outcome as compared to our data might be due to a more extensive disease treated in our cohort (for eg only two of our patients’ had a chest wall reirradiation only). In our group of patients re-irradiation was given with a median dose of 40 Gy to the PTV. Reirradiation was combined with concomitant chemotherapy in nine cases. In our experience concurrent chemotherapy with reirradiation is feasible in breast cancer without higher acute toxicity. We chose mostly capecitabine as concurrent chemotherapy regimen because of the high clinical experience in combination with radiotherapy treatment.

There are no recommendations regarding DVH data and normal tissue complication probabilities when very large breast tumors are treated with IMRT, VMAT or tomotherapy. For most of our analyzed patients, we recommended constraints based on Emami et al. [9]. Few patients were treated after 2010 with constraints based on QUANTEC [26]. The V20 and V30 are within the known constraints; however the V5 in our patients is fairly high as compared to the up to date recommendations. These deviations from internal and external standard operation procedures (SOP) is predominantly due to patients’ anatomy as well as complexity of the target volumes, and were one of the indications why tomotherapy was chosen to treat the patients. Generally, dose constraints as well as accepted tolerances are individual decisions made for every single patient, and weighing of target coverage, applied dose and sparing of normal tissue has to be done thoroughly. Due to the retrospective nature of the study, no prospective long-term follow-up data is available; currently, long-term observations are in process to assess the long-term toxicites in these patients. One recent publication assessed nine breast cancer patients with a mean followup of 10.3 months [27]. A longer follow-up was available in 6 patients and the authors reported a pneumonitis in one of the 6 patients. This patient had a V20 of 25% and V5 of 64% and a preexisting lung disease. Nonetheless, the lung V20 for all patients ranged from 25 to 35% (mean 29%) and the V5 for all patients ranged from 51 to 75% (mean 66%); thus an exclusively dosimetric explanation could not be offered by the authors [27]. Further studies are needed on this topic.

## Conclusion

HT is feasible for locally advanced and recurrent thorax embracing breast cancers with acceptable acute toxicity and a good therapy effect. Depending on the volume size, complexity as well as the individual patient’s anatomy, the choice of helical IMRT has to be made and can lead to improved treatments plans with convincing outcome.
